# Child temperament, parent emotions, and perceptions of the child’s feeding experience

**DOI:** 10.1186/1479-5868-9-64

**Published:** 2012-05-29

**Authors:** Sheryl O Hughes, Richard M Shewchuk

**Affiliations:** 1 USDA/ARS Children’s Nutrition Research Center, Department of Pediatrics, Baylor College of Medicine, 1100 Bates Street, Houston, TX, 77030-2600, USA; 2 Department of Health Services Administration, University of Alabama at Birmingham, 560 Webb Building, 1530 3rd Avenue South, Birmingham, AL, 35294-3361, USA

## Abstract

**Background:**

Associations between parent and child characteristics and how they influence the approach parents take toward children in the feeding environment have not been examined extensively, especially in low-income minority families who are at a higher risk for obesity. The primary aim of the study was to examine positive and negative parent emotions as potential mediators of the relationship between child temperament and parents’ perceptions of strategy effectiveness and problems encountered in feeding children fruit and vegetables.

**Methods:**

Participants were low-income families (n = 639, 73% minority, children aged 3–5 years) participating in Head Start programs in two states. Parents completed the Children’s Behavior Questionnaire (CBQ), Positive and Negative Affect Schedule (PANAS), and measures of strategy effectiveness (teachable moments, practical methods, restriction, and enhanced availability) and problems encountered (vegetable characteristics, child attributions for dislike, external influences, and parental demands) in feeding children fruit and vegetables.

**Results:**

Positive parent emotions partially mediated the relationship between child Effortful Control and strategy effectiveness and fully mediated the relationship between Surgency and strategy effectiveness. Although negative parent emotions were associated with increased perception of problems in feeding children fruit and vegetables, the relationship between Negative Affectivity and problems in feeding was partially mediated by negative parent emotions.

**Conclusions:**

Positive parent emotions facilitated perceived effectiveness of feeding strategies, with child Effortful Control and Surgency instrumental to this process. Understanding mechanisms in parent–child feeding is important when developing interventions designed to promote healthy child eating behaviors.

## Introduction

Healthy dietary patterns that include high intakes of fruit and vegetables have been associated with decreased risk for obesity [1], cardiovascular disease [2], and some types of cancer [3]. However, our understanding of the social contexts that promote healthy dietary patterns in children are not well understood. One developmentally important context that may have enduring consequences for child health outcomes is the family environment. Research in this area has tended to focus on parent behavioral strategies that are used to modify children’s dietary intake - specifically, those focused on behaviors that are intended to get the child to eat more or to get the child to eat less (i.e., pressure to eat, restriction, and monitoring) [4]. These previous studies have not captured the wide range of potential strategies that may be used by parents to encourage healthy eating in their children. More importantly, few studies have examined the shared dynamic that exists between the parent and child in the feeding environment and the nature of those interactions. Only recently have parenting researchers begun to take into consideration the characteristics of the child and the emotional state of the parents and how they might influence parents’ view of the social environment including perceptions of the strategies and problems encountered in their shared environment [5]. To this end, the interplay between parent and child characteristics and the influence these constructs have on the cognitive appraisals that parents have about their interactions with the child in the eating environment have not been examined extensively. This is especially true in low-income ethnic minority families with a higher risk for obesity.

### Child temperament and parenting

Children make important contributions to their social interactions; therefore, characteristics of the child should be considered when examining the shared feeding environment [6]. Temperament plays an important role in the parent–child dynamic because parents may react differently to children who are more internally controlled as compared to those who are more reactive in their temperaments [7]. An incompatible or “poor fit” between what parents expect from their children and the temperament of their child may lead to stressful interactions whereas a “good fit” may lead to mutually pleasurable interactions. Prior literature suggests that parents who have “difficult” children report more negative emotions and are more likely to respond with force compared to parents with less difficult children [8,9]. It has been posited that parents may use food as a means of soothing their children during stressful interactions around food [10]. Essentially, the fit between the temperament of the child and parents’ expectations may impact how the feeding environment is viewed [11,12].

### Parent emotions and perceptions of the shared environment

As a key element in motivational processes involving parenting [13], parents’ emotions are thought to impact their intentions to attend to and support their children [14]. Positive parent emotions provide a supportive context for children’s development [15] and have been associated with more child-oriented concerns and supportive behavior by parents [14]. Similarly, positive parent emotions facilitate the ability for children to deal with stress and adversity [16] and maintain task motivation [17]. In contrast, negative parent emotions, such as anger, sadness, and guilt, provide a hostile and critical context for children [18], and appear to interfere with parenting by reducing parents’ ability to implement developmentally appropriate parenting strategies [19]. Within the parent–child dyad, negative parent emotions have been shown to promote disruptive behavior among children, especially in low-income families [20]. Overwhelming evidence exists that support the influence of parent emotions on child outcomes [13,14]; however, little research has focused on parent emotions in the shared parent–child feeding environment.

### The present study: An integrated model

As shown, previous studies have examined pieces of the overall parent–child dynamic (i.e., how child temperament and parent emotions have influenced parenting separately); however, little research has focused on a more integrated model of parent and child characteristics around parenting/feeding. We proposed to gain a deeper understanding of these relationships by more carefully examining how child temperament might influence parent’s appraisal of the feeding environment after being filtered through the emotional state of the parent. Therefore, the present study examined relationships between child temperament, parent emotions, and various strategies and problems encountered with children in the feeding environment with the specific aim of investigating how parents’ emotional states influence the perception of various strategies and problems encountered in getting their children to eat fruit and vegetables. It was hypothesized that parents reporting more positive emotions would perceive strategies as more effective and problems as less of a barrier. Conversely, it was hypothesized that parents reporting more negative emotions would perceive strategies as less effective and problems as more of a barrier. Moreover, we hypothesized that parents’ emotions would mediate the effects that child temperament would have on parents’ perceptions of strategies and problems encountered in attempting to get children to eat fruit and vegetables.

## Methods

### Participants

Participants (Table 1) were part of a larger study to investigate facilitators and barriers to fruit and vegetable intake among preschool children. Participants included 639 primary caregivers (n = 282 African-American, 185 Hispanic, and 172 white) whose children were enrolled in Head Start facilities across two sites (Alabama, and Houston, Texas). The primary caregiver was defined as the person most often responsible for what the child ate outside of preschool (92.7% mothers, 6.3% grandmothers, 1% other). Children ranged from three to five years old and were 48% female and 52% male. All participants were eligible for Head Start, which serves a population whose family income is equal to or below the federal poverty level. The study was reviewed and approved by the Institutional Review Boards at Baylor College of Medicine and the University of Alabama at Birmingham. Written consent was obtained from the parents who particpated in the study.

**Table 1:** Participant Characteristics

	**N = 639**
Ethnicity	
White	27.4%
Hispanic	28.7%
African-American	43.6%
Parent gender - female	95.0%
Child gender - female	47.7%
Age, mean in years (SD)	
Parent	31.6 (8.1)
Child	4.5 (0.6)
Education of parent	
High school diploma or less	58.2%
Some college or more	36.2%
Marital status, parent married	48.2%
Parent BMI	
Underweight (BMI < 18.5)	1.1%
Normal (18.5 BMI < 25)	20.9%
Overweight (25 BMI < 30)	28.3%
Obese (BMI 30)	49.6%
Child BMI^a^	
Underweight or at risk (15^th^ percentile)	3.6%
Normal (>15^th^ to <85^th^ percentile)	56.6%
Overweight or obese (85^th^ percentile)	39.8%

^a^From age- and gender-specific cut points of the CDC growth charts.

### Procedures

Qualitative work was conducted at the beginning of the study to identify commonly used strategies and problems encountered by parents when attempting to get children to eat heathly foods. Sixteen structured focus group meetings were conducted with Head Start parents (8 strategies; 8 problems) using the nominal group technique (8 – 10 parents per meeting). The meetings were equally distributed among African-American, Hispanic, and white parents. The nominal group technique (NGT) is unlike a traditional focus group in that it elicits responses to a single question – in this case “What are the ways parents can help their preschool child eat healthy foods?” for strategies and “What are the problems parents face with having their preschool child eat healthy foods?” for problems. The structured format of the NGT meeting minimizes discussion not pertinent to the topic and provides concise responses that are prioitized by each participant. The collective rank ordering of responses therefore provided an objective view of how parents perceived the importance of strategies and problems in the context of encouraging children to eat healthy foods. Prioritized responses from each of the NGT meetings were aggregated and used as the basis for developing a set of card sort tasks and rating scales for further assessment of strategies and problems.

The card sort tasks were used with a second group of 761 Head Start parents in an interview format who did not participate in the earlier qualitative work to better understand how the strategies and problems were cognitively organized. This resulted in the development of the Feeding Strategies Scale [21] and the Feeding Problems Scale. Of this group, 639 parents provided complete data for all of the variables used in this study. Analyses revealed no significant differences between parents providing complete and incomplete data. Although the Feeding Strategies Scale has been used in previous research [21], this is the first time the Feeding Problems Scale has been used in a study.

### Measures

Participants completed the Q-sort and rating scales through interviews. The same parents also completed other parent-reported measures and returned them to the Head Start centers in sealed envelopes.

#### Parent feeding strategies and problems

A modified version of the 33-item Feeding Strategies Scale (FSS) [21] and the 26-item Feeding Problems Scale (FPS) were used to measure parent perceptions in feeding children fruit and vegetables. Items are scored on a 3-point scale to reflect current level of perceived effectiveness of feeding strategies (1 – “not very effective”, 2 – “a little bit effective”, 3 - “very effective”) and feeding problems (1 – “not a problem”, 2 – “a little problem, 3 - “a big problem”) in getting children to eat fruit and vegetables. Items for these measures were generated through nominal group techniques, Q-sort procedures, multidimensional scaling, and clustering techniques. The modified version of the FSS used in this study included four subscales: 1) Teachable Moments, e.g., “use mealtime to teach child about healthy eating,” 2) Practical Methods, e.g., “use fun shapes,” “mix F or V with other foods,” 3) Restriction of Junk Foods/Sweets, e.g., “limit snacking,” “limit sweet drinks,” and 4) Enhanced Availability of F & V, e.g., “place where child can easily reach,” “include in most meals.” The Firm Discipline subscale was not included as an indicator of the strategies latent variable in our model because conceptually this subscale could be construed as being substantively different in how parents interacted with their children in the context of feeding. Specifically, the included indicators (Teachable Moments; Practical Methods; Restriction of Junk Foods/Sweets; Enhanced Availability) measured a more proactive parent approach in helping children to eat fruit and vegetables whereas the Firm Discipline subscale was viewed as being more reactive and punitive.

The FPS includes four subscales: 1) Unappealing Characteristics of Fruit/Vegetable, e.g., “will not eat vegetables because of their smell,” 2) Child Attributions for Dislike, e.g., “likes fast food better than F & V,” 3) External Influences on Child Dietary Behavior, e.g., “other people give child junk food,” and 4) Competing Parental Demands, e.g., “too busy to cook.”

#### Positive and negative affect schedule (PANAS)

The PANAS [22] includes two 10-item scales measuring two distinct subscales – Positive Affect which measures positive emotions (e.g., interest, determination, enthusiasm and pride) and Negative Affect which measures negative emotions (e.g., fear, distress, hostility and shame). Items are scored on a 5-point scale (1 – “very slightly or not at all” to 5 – “extremely”), and scores range from 10 to 50, with higher scores on Positive Affect indicating more positive emotions and higher scores on Negative Affect indicating more negative emotions. Good internal and test–retest reliability have been shown in large samples and construct validity demonstrated by correlations with other existing multi-affect and emotion measures [22]. In the current sample, coefficient alpha was .86 for positive parent emotions (Positive Affect) and .86 for negative parent emotions (Negative Affect), with a scale intercorrelation of - 0.03.

#### Children’s behavior questionnaire (CBQ very short form)

The Children’s Behavior Questionnaire was developed to assess child temperament, defined as constitutionally based, individual differences in reactivity and self-regulation [23]. Constitution is defined as the individual’s relatively enduring biological make-up, influenced over time by heredity, maturation, and experience. Structural analyses of the CBQ scales reveal good construct validity and consistently result in three broad factors of Negative Affectivity, Effortful Control and Surgency-Extraversion. The factor structure remains consistent across age groups and cultures. Evidence for convergent validity comes from a number of sources, including parent agreement and prediction of social and laboratory behavior patterns [23]. The subscales comprising the CBQ Very Short Form [24] are consistent with the three factors from the long form. Recent data with the short form has demonstrated acceptable internal consistency for all samples including low-income ethnic minorities and confirmatory factor analyses indicated an adequate fit for the three-factor model [24]. The Negative Affectivity subscale has high positive loadings on Sadness, Fear, Anger/Frustration, and Discomfort (CBQ long form) and is conceptually similar to Neuroticism in adults (e.g. ‘gets angry when s/he can’t find something s/he wants to play with’). The Effortful Control subscale has high positive loadings for Inhibitory Control, Attentional Control, and Low Intensity Pleasure (CBQ long form) and is similar to Conscientiousness/Constraint in adults (e.g. ‘prefers quiet activities to active games’). The Surgency subscale is characterized by high positive loadings on Impulsivity, High Intensity Pleasure, and Activity Level (CBQ long form) and is similar to Extraversion in adults. Caregivers are asked to consider their child’s reaction in the past six months to 36 situations (e.g., “prefers quiet activities to active games,” “gets angry when s/he can’t find something s/he wants to play with”) and respond using a 7-point response scale ranging from “extremely untrue” to “extremely true.” In the current sample, coefficient alpha was .65 for Negative Affectivity, .74 for Effortful Control, and .63 for Surgency-Extraversion.

#### Anthropometrics

Parent and child height and weight were measured (light clothing, without shoes) by our staff in duplicate. These scores were averaged and used to compute child and parent BMI (kg/m^2^). For adults, overweight was defined as BMI of 25.0 to 29.9 and obese as BMI ≥ 30.0, according to the World Health Organization criteria [25]. For children, BMI *z*-scores and percentiles were calculated using age- and gender-specific cut points [26]. Overweight was defined as at or above the 85th percentile but less than the 95th percentile, and obese as BMI ≥ 95th percentile [27].

### Statistical analyses

Path analysis with latent variables was used to test the hypothesized model [28]. Structural equation modeling enables the evaluation of multivariate path models, allowing the simultaneous evaluation of the direct, indirect, and total effects of a set of predictor variables on multiple mediators and multiple outcome variables. The model included three exogenous, observed variables (the three factor scores from the CBQ), parent emotions as mediator variables (the two subscales from the PANAS used as two latent variables with single indicators, loadings fixed at 1 and errors fixed at 0), and parent feeding strategies and problems as dependent variables (used as two latent variables, each with four factor scores from the FSS and FPS as indicators).

The robust maximum likelihood (RML) estimation method was used to generate the standardized parameter estimates for the structural equation models, since the data were not multivariate normal. PRELIS, the pre-processing program of LISREL, estimated the asymptotic covariance matrix of the sample variances and covariances that the RML method requires. The model fit was evaluated using the Satorra-Bentler (S-B) scaled chi-square statistic [29] in order to correct for nonnormality. A chi-square that is not significant (*p* > .05) indicates a good fit, as the model does not differ significantly from the observed data. Since significant chi-squares that reject the model can occur even when the model fit is relatively good, other fit indices were also used to evaluate the models. These included the comparative fit index (CFI), Non-Normed fit index (NNFI), Standardized Root Mean Squared Residual (SRMR), and the root mean square error of approximation (RMSEA). RMSEA values of 0.08 and 0.06 or less, SRMR values of less than .08, and CFI and NNFI values of .90 and .95 or higher indicate an acceptably close and an excellent fit [30]. Chi-square difference tests, using Satorra-Bentler’s correction factor [31], were used to compare the hypothesized models with alternative models in which direct paths were added, since the hypothesized and alternative models were nested.

A direct effects model that contained all direct and indirect paths from child temperament to the mediators and behavioral outcomes was estimated in addition to the hypothesized mediation model of only indirect effects to examine directs effects. Tests of mediation for specific mediators, controlling for all other variables in the model, followed the procedure of Baron and Kenny [32]. Mediation was inferred when (1) the direct effect between the independent variable and the mediator was significant, (2) the direct effect between the mediator and the outcome variable was significant, and (3) the direct effect of the independent on the outcome variable, with the mediator in the model, was significantly reduced as determined by the Aroian version of the Sobel test [32,33] using the unstandardized coefficients and standard errors from the all-paths model (i.e., *z* ≥ 1.96).

## Results

**Figure 1:**
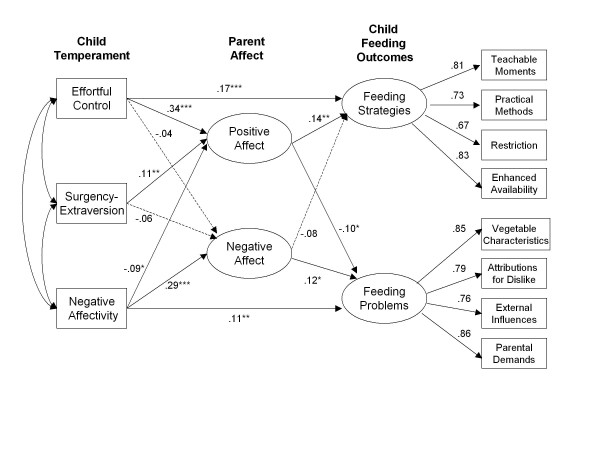
Final structural model showing direct and indirect relationships between child temperament and parent strategies and problems in feeding fruit and vegetables to children.

Means, standard deviations, and zero-order correlations for the study variables are shown in Table 2. Results of the hypothesized model revealed an adequate fit to the data, S-B χ^2^(57, N = 639) = 240.76, *p* < .01, RMSEA = .071 (0.062 - .080), NNFI = .93, CFI = .95, SRMR = .069. However, modification indices and the direct effects model indicated that the direct effect of child Effortful Control on parent feeding Strategies was still significant, as well as the direct effect of child Negative Affectivity on parent perceived feeding Problems, so these paths were added to the hypothesized model one at a time (Child Effortful Control → Strategies, Δ df = 1, Δ χ_S-B_^2^ = 7.00, p < .01; Child Negative Affectivity → Problems, Δ df = 1, Δ χ_S-B_^2^ = 8.64, *p* < .01) for a resulting model fit of S-B χ^2^(55, N = 639) = 215.86, p < .01, RMSEA = .068 (0.058 - .077), NNFI = .94, CFI = .96, SRMR = .063. In the final model (Figure 1), each dimension of child temperament was significantly related to parent positive emotions, such that higher levels of child Effortful Control and Surgency were associated with increased positive emotions in parent, while increased child Negative Affectivity was associated with decreased positive emotions. Only one aspect of child temperament, Negative Affectivity, was significantly related to parent negative emotions. Among the hypothesized mediators, parent positive emotions were positively related to parent feeding strategies (β = .14, *p* < .01) and negatively related to problems (β = −.10, *p* < .05), indicating an increase in effectiveness ratings for feeding strategies and a decrease in perceptions of feeding problems with increased positive emotions in parent. In contrast, parent negative emotions were negatively, but not significantly, related to parent feeding strategies (β = −.08, t = −1.89) and positively related to feeding problems (β = .12, p < .05), indicating decreased perceived effectiveness of various feeding strategies and an increase in perception of feeding problems.

**Table 2:** Variable Pearson Correlations, Means, and Standard Deviations

**Variable**	**1**	**2**	**3**	**4**	**5**	**6**	**7**	**8**	**9**	**10**	**11**	**12**	**13**
Child Temperament													
1. Effortful control	1.00												
2. Surgency-extraversion	.09	1.00											
3. Negative affectivity	.15	.03	1.00										
Parent Emotions													
4. Positive	.33	.15	-.04	1.00									
5. Negative	-.00	-.07	.29	-.01	1.00								
Parent Feeding Strategies													
6. Teachable moments	.22	.02	.02	.17	-.06	1.00							
7. Practical methods	.15	.05	.01	.11	-.01	.60	1.00						
8. Restriction of junk foods/sweets	.14	.04	-.04	.12	-.04	.50	.53	1.00					
9. Enhanced Availability	.15	.03	-.07	.18	-.11	.68	.58	.57	1.00				
Parent Feeding Problems													
10. Unappealing Characteristics	-.08	-.04	.14	-.14	.13	-.18	-.04	-.11	-.32	1.00			
11. Child attributions for dislike	-.05	-.04	.14	-.04	.13	-.15	-.00	-.14	-.27	.72	1.00		
12. External influences	-.04	-.07	.12	-.08	.12	-.07	-.02	-.13	-.15	.62	.56	1.00	
13. Competing parental demands	-.05	-.08	-.08	-.07	.12	-.13	-.06	-.12	-.23	.71	.65	.71	1.00
Mean	5.4	4.6	4.4	35.4	20.1	2.4	2.5	2.4	2.6	1.6	1.7	1.5	1.5
Range	2.4 - 7.0	1.8 - 6.7	1.1 - 6.8	12 - 50	10 - 50	1 - 3	1 - 3	1 - 3	1 - 3	1 – 2.9	1 - 3	1 - 3	1 - 3
Standard Deviation	0.7	0.7	0.8	7.7	7.3	0.4	0.3	0.4	0.4	0.5	0.6	0.4	0.4

All coefficients with an absolute value of .08 or greater are significant at .05.

For mediation effects of parent emotions, three paths for the strategies outcome and three for the problems outcome met the criteria for mediation testing (Table 3). Parent positive emotions significantly mediated both child Effortful Control and Surgency for the strategies outcome, supporting the hypothesized influence of child temperament on feeding strategies by facilitating parents’ positive emotions. Since the direct path from Effortful Control to strategies was also significant, this indicated that there were both significant direct and indirect effects for child Effortful Control on parent beliefs about the effectiveness of feeding strategies. Likewise, for child Negative Affectivity, there were also significant direct and indirect effects on the feeding problems outcome. In contrast, parent positive emotions fully mediated the relation between child Surgency and parent feeding strategies, since no direct paths remained significant in the model. The magnitude of the mediated effects was stronger for positive emotions, specifically for the Effortful Control pathway (child EffCon → parent Pos → Strategies: .34 x .14 = .05; child Surgency → parent Pos → Strategies: .11 x .14 = .02), than for negative emotions (child Neg → parent Neg → Problems: .29 x .12 = .03).

**Table 3:** Mediation Tests for Specific Indirect Effects of Child Temperament

**Path**	**Z**^**a**^	**p**
Strategies		
Child Effortful Control - > Parent Pos Emotion - > Strategies	2.67	.008
Child Surgency - > Parent Pos Emotion - > Strategies	2.05	.040
Child Neg Affect - > Parent Pos Emotion - > Strategies	−1.67	.096 ns
Problems		
Child Effortful Control - > Parent Pos Emotion - > Problems	−1.71	.087 ns
Child Surgency - > Parent Pos Emotion - > Problems	−1.48	.139 ns
Child Neg Affect - > Parent Neg Emotion - > Problems	2.09	.037

^a^ z-value for Aroian version of the Sobel test.

## Discussion

Our research extends previous feeding studies by examining the role of child temperament and parent emotions as they relate to parents’ perceptions of the effectiveness of various feeding strategies and problems encountered when feeding their children. Our results show that parents’ positive emotions were associated with higher levels of perceived feeding strategy effectiveness and negatively related to parents’ perceived seriousness of problems in feeding their children fruit and vegetables. Parents’ negative emotions were directly related to the problems they perceived to have when feeding their children fruit and vegetables. Further, we observed that dimensions of child temperament, specifically Effortful Control and Surgency, were indirectly related to parents’ perceptions of feeding strategy effectiveness, and in the case of Effortful Control, also directly related to these perceptions. In addition, a third dimension of child temperament, Negative Affectivity, was directly related to parents’ negative emotions and directly and indirectly related to parents’ perceptions of more problems in feeding their children.

Our results emphasize the importance of both parent and child characteristics in feeding children, and are consistent with an ecological parenting framework [34,35]. Both constitutionally based individual differences in children’s reactivity and self-regulation [36], and parent emotions were linked to parent feeding strategies and problems in our sample. Our work extends the growing body of literature linking child temperament to child eating behaviors. A recent study showed that children (aged 3 to 8) with more emotional temperaments were reported by their mothers to display more food avoidant eating [37]. Other studies have linked child temperament and mothers’ mental health to feeding in very young children [38], but this is the first study that has focused specifically on positive and negative emotions in parents and tested it as a mediator of child temperament.

Our study also extends a growing body of research on the differential effects of positive and negative emotions on cognitive processes and task performance. Specifically, our findings regarding the link between positive parent emotions and their beliefs about feeding strategy effectiveness support previous research on the influence of positive emotions on expectancy motivation [39]. Erez and Isen showed that positive emotions affected the three components of expectancy theory [39], enhancing expectancy (effort and performance), instrumentality (performance and outcome), and valence (outcome is desirable). Our research extends this work by demonstrating the effects of positive emotions on performance expectancies within the parent–child feeding environment. Likewise, our observation of the negative relationship between positive parent emotions and problems in feeding children fruit and vegetables suggest that positive emotions may serve as a buffer when confronting challenges associated with parent–child feeding [40]. Both child Effortful Control, a core aspect of self-regulation that serves to monitor and control thoughts and actions, and child Surgency, a dimension of child temperament that reflects positive anticipation, activity level, and sensation seeking, were directly associated with positive parent emotions. Having children who exhibit positivity, social skill, and playfulness, who may be more willing and able to control behavior, focus attention, follow parent requests, and, who are, in short, pleasant to be with, likely is an important source of positive emotion for parents. This source of positive emotions may serve to broaden parents’ ‘thought-action repertoires’ regarding what types of feeding strategies work for their children [41].

In contrast to the assumed facilitative effects of positive emotions, the effects of negative emotions on parents’ perceptions of the feeding experience would appear to act as an impediment. There is extensive evidence that negative emotions, both acute and chronic, impede social functioning, cognitive processing, and goal-relevant behaviors in both adults and children [42,43]. There also is evidence that children’s negative emotions are associated with mothers’ negative emotions as they relate to parent–child feeding interactions [44]. Moreover, we found that negative emotions in parents and in children were associated with the seriousness of perceived feeding problems.

It is important to acknowledge several limitations to this work. First, the present study results are cross-sectional and require caution in regard to causal inferences. Although we present a theoretically plausible model, given that the constructs of interest have not been examined before in the same context, our structural equation model should be viewed as only descriptive and exploratory in nature – not as a confirmatory or causal analysis. This is particularly imporatant in considering that we were not able to assess the temporal ordering or omitted variable assumptions that are essential to reach conclusions regarding mediated causal ordering. It is concievable that there are potential confounding variables that, if included in the model, could result in a very different pattern of derived coefficients and conclusions [45,46]. Furthermore, longitudinal and intervention studies would be helpful in clarifying the nature of the relationships among the constructs that were considered in our study.

Additionally, we measured parent expectations regarding feeding strategy effectiveness, not their actual use. Importantly, it would be informative to conduct studies to address how the child temperament and parent emotion dynamic affects the use of strategies, the nature of the problems encountered and in turn, children’s eating behaviors and weight status. Generalizability of the results may also be a concern, since the sample population was comprised of only low-income parents of preschool children.

In conclusion, our results support a model in which two motivational factors important to parent–child feeding (parent perceptions about feeding strategy effectiveness and problems involved in feeding children healthy foods) are directly linked to child temperament and parent emotions. Positive parent emotions mediated child temperament and were associated with lower levels of perceived problems in getting children to eat fruit and vegetables. Regardless of what children are like, positive parent emotions appear crucial to the development of healthy eating habits in children. Understanding the beneficial effects of positive emotions and incorporating them into intervention efforts may be essential to improving eating behaviors in children.

Therefore, future intervention efforts should consider approaches aimed at helping parents become more aware of their own emotions and how these may be influenced by their children’s temperament when assessing the effectiveness of various feeding strategies and the nature of problems encountered in the feeding domain. Future interventions also might focus on helping parents to be realistic in their appraisal of what is happening in the feeding domain. Parents would benefit from problem solving approaches to learn what works and what does not work, and what actually constitutes a problem that can interfere with the parent–child feeding process.

## Competing interests

The authors declare that they have no competing interests.

## Authors’ contributions

SOH, RMS made substantial contributions to the conception, design, acquisition, analysis and interpretation of the data. SOH, RMS have been involved in drafting the manuscript and revising it critically for important intellectual content. SOH, RMS have given final approval of the version to be published.
